# Bioanalytical considerations for quantification of ondansetron in rat microdialysis study using LC-MS/MS

**DOI:** 10.1016/j.microc.2025.113082

**Published:** 2025-02-18

**Authors:** Yae Eun Chong, Anna Siemiątkowska, Christine Yohn, Simon Haroutounian, Leonid Kagan, Katarzyna Kosicka-Noworzyń

**Affiliations:** a Department of Pharmaceutics and Center of Excellence for Pharmaceutical Translational Research and Education, Ernest Mario School of Pharmacy, Rutgers, The State University of New Jersey, 160 Frelinghuysen Road, Piscataway, NJ 08854, USA; b Department of Physical Pharmacy and Pharmacokinetics, Poznan University of Medical Sciences, 3 Rokietnicka Street, 60-806 Poznań, Poland; c Department of Anesthesiology and Washington University Pain Center, Washington University School of Medicine, St Louis, MO 63110, USA

**Keywords:** Method validation, Tissue concentration, Pharmacokinetics, Drug distribution

## Abstract

**Background:**

Ondansetron is an anti-emetic drug and has been recently identified as a therapeutic for neuropathic pain. Permeability of ondansetron into the nervous system may be impacted by the presence of P-glycoprotein (Pgp) at the blood brain barrier, since ondansetron is a substrate of Pgp, which means it may not easily cross into the brain. In order to determine a drug concentration within the brain, microdialysis can be used, which measures unbound analyte concentrations in tissues or body fluids.

**Aim:**

The purpose of this study was to develop and validate an LC-MS/MS method to measure ondansetron concentrations in serum and brain microdialysates for pharmacokinetic studies in rats. To the best of our knowledge, a fully validated method for the quantification of ondansetron in brain microdialysis studies has not been reported so far.

**Methods:**

Chromatographic separation was achieved at a flow rate of 0.6 mL/min using the Gemini C18 column (5 μm; 50 × 4.6 mm) and a mobile phase consisting of 10 mM ammonium formate with 0.1 % formic acid in water and 0.1 % formic acid in acetonitrile (55:45, v/v). Serum samples were prepared by protein precipitation; for microdialysate, no specific cleaning procedure was needed, they were only diluted with the internal standard solution.

**Results:**

The presented method requires only a small volume of serum (2.5 μL) and microdialysate (15–20 μL), which allows for frequent sampling in animals. Moreover, it enables high throughput due to a short run time and straightforward sample preparation. Artificial cerebrospinal fluid (aCSF) was confirmed to be useful as a surrogate matrix for brain microdialysate. The lower limit of quantitation for ondansetron was 0.01 μg/mL for serum and 0.025 ng/mL for microdialysate. A significant ion suppression for ondansetron was observed in brain microdialysate and aCSF, but the IS-normalized matrix factor was close to 1.0.

**Conclusion:**

The developed LC-MS/MS method met the FDA and EMA validation criteria and was successfully applied to the in vivo pilot pharmacokinetic study. It was sensitive enough to capture the low ondansetron concentrations in brain microdialysate and universal enough to measure high ondansetron concentrations in rat serum.

## 1. Introduction

Ondansetron is a 5-HT_3_ receptor antagonist that is under investigation for the treatment of neuropathic pain [1]. However, the P-glycoprotein (Pgp) at the blood–brain barrier might limit its exposure in the central nervous system and thus reduce its efficacy [2]. Our previous study with Pgp-knockout rats has demonstrated that a lack of Pgp enhanced ondansetron permeability into the brain [3]. The study involved the determination of ondansetron concentrations in rat whole brain, spinal cord, and cerebrospinal fluid (CSF). While this recent study of ours indicated the action by which ondansetron may cross into the brain and thus the nervous system, it failed to measure the unbound drug concentration within the brain.

The free (unbound) form of a drug is the most relevant to its efficacy, and this form can be directly measured at the site of action by microdialysis [4]. The technique measures the time course of the free drug in a tissue of interest by using a semi-permeable probe [5]. Analytes diffuse across the semi-permeable membrane down their concentration gradient, and dialysates are collected for analysis [6]. While this technique is very powerful in characterizing unbound drug concentrations within the brain and other target tissues, there are limitations that should be considered when designing a microdialysis study to ensure accurate analyte concentrations are collected. There are several aspects to the microdialysis setup and application that can impact the microdialysates collected drug concentration. For instance, drugs, especially lipophilic drugs, may bind to and be absorbed by the microdialysis tubing and probe materials [5]. Determining how the drug interacts with the materials used to collect microdialysates is needed since the volume of samples collected in this process are small and may contain very low drug concentrations. On the other hand, sophisticated clean-up procedures are usually not necessary prior to chromatographic separation since the matrix composition is simpler than matrices containing proteins (e.g., plasma). Nevertheless, each bioanalytical method needs to be properly validated to ensure that the determined analyte concentrations in a specific matrix are reliable. Still, in most microdialysis studies involving LC-MS/MS, validation results were not presented [7,8], or only few selected parameters, such as linearity, accuracy, and precision, were evaluated [9,10]. These reports omitted the important tests required by the regulatory agencies (European Medicines Agency, EMA [11] and Food and Drug Administration, FDA [12]). For example, some of these published studies did not exclude the presence of matrix effect, which is a very common occurrence in mass spectrometric assays that can seriously affect the method’s performance [13,14]. They also did not indicate the analytes’ adsorption to laboratory plasticware or glassware that could occur in microdialysates since the matrix has almost no protein content [10,13,14]. We have previously reported challenges with the quantification of tariquidar in another low-protein matrix, CSF [15]. Despite acceptable linearity, precision, and accuracy, the method could not be successfully validated due to the significant adsorption of the analyte to the collection tubes.

In the literature, multiple bioanalytical methods exist for the quantification of ondansetron in various matrices, such as plasma (e.g., human [16–23], or rat [16,24,25]), CSF [17] and tissue homogenates [25]. The two assays reported for measuring ondansetron in microdialysates using LC-MS/MS [26] or HPLC-UV [27] presented no validation data, which questions their reliability. Also, despite many existing methods for ondansetron quantification, no published assay met the specific requirements for this microdialysis study. Specifically, an assay that could handle very low sample volumes (preferably, below 15–20 μL), achieve a high sensitivity (due to the expected low drug levels in microdialysate), and cover a wide calibration range if a correlation between serum and tissue concentrations is to be analyzed. Conversely, the sample volume required for the published methods usually ranged from 25 μL to 1000 μL [18–23], which would not be practical in a microdialysis study. Most published methods also reported a narrow calibration range and insufficient sensitivity [18–21].

Our study aimed to develop and validate a robust LC-MS/MS method to quantify ondansetron in rat brain microdialysates and serum. To the best of our knowledge, a fully validated method for the quantification of ondansetron in brain microdialysis studies has not been reported so far. The presented method has a high sensitivity and requires a low sample volume, which allows for frequent sampling to obtain a full pharmacokinetic profile. Moreover, the method enables high throughput due to the straightforward sample preparation (simple protein precipitation for serum and no clean-up procedure for microdialysate) and very short run time. The developed method was applied to an intracerebral microdialysis study in rats to evaluate ondansetron pharmacokinetics in the blood and brain.

## 2. Methods

### 2.1. Materials and methods

#### 2.1.1. Chemicals and Reagents

Ondansetron hydrochloride dihydrate salt (purity > 99 %) was purchased from Sigma Aldrich (St. Louis, MO, UA), and isotope-labeled ondansetron, ondansetron-D_3_ (chemical purity 98 %, isotopic purity 99.9 %), was obtained from Toronto Research Chemicals (North York, ON, Canada). Pooled male rat serum was obtained from BioIVT (Westbury, NY, USA). Artificial CSF (aCSF) was from Harvard Apparatus (Holliston, MA, USA) and its composition was the following (mM): Na 150, K 3.0, Ca 1.4, Mg 0.8, P 1.0, Cl 155; pH = 7.2. Formic acid (≥99 % purity, LCMS grade) was purchased from Fisher Scientific (Fair Lawn, NJ, USA) and ammonium formate (≥99 % purity, LCMS grade) was obtained from Honeywell (Morristown, NJ, USA). Other solvents (acetonitrile, methanol, isopropanol, water) were of LCMS grade and were purchased from either Sigma Aldrich (St. Louis, MO, USA) or Fisher Scientific (Fair Lawn, NJ, USA).

#### 2.1.2. Stock and working solutions

Stock solutions for ondansetron (0.25 mg/mL, free base) and ondansetron-D_3_ (0.10 mg/mL) were prepared in acetonitrile. The working solutions for ondansetron were prepared by diluting the stock solutions with acetonitrile to obtain the concentrations of 0.10 – 250 μg/mL for serum and 0.25 – 500 ng/mL for microdialysates. The internal standard (IS) working solutions were prepared in acetonitrile at 100 ng/mL for serum and 1 ng/mL for microdialysates. The stock and working solutions were stored at −20 °C.

#### 2.1.3. Calibration standards and quality control samples

The calibration standards for serum were prepared by 10-fold dilution of the working solutions with a blank rat serum to obtain a calibration curve of 0.01 – 25 μg/mL. For microdialysate, the working solutions were diluted 10 times with aCSF, resulting in a calibration curve of 0.025 – 50 ng/mL. The quality control (QC) samples were prepared in the same manner to obtain four levels at 0.01 μg/mL (lower limit of quantification, LLOQ), 0.025 μg/mL (low-QC), 0.5 μg/mL (mid-QC), 25 μg/mL (high-QC) for serum and 0.025 ng/mL (LLOQ), 0.05 ng/mL (low-QC), 1 ng/mL (mid-QC), 50 ng/mL (high-QC) for microdialysates. The calibration standard and QC samples were further processed using the protocol described below for the study samples.

#### 2.1.4. Sample preparation

For serum analysis, 2.5 μL of serum was precipitated with 100 μL of IS solution. The samples were vortexed for 10 s, shaken for 10 min, and centrifuged for 10 min at 15,700*g* at 4 °C. Fifteen μL of the supernatant were transferred to the HPLC vials containing 485 μL of acetonitrile, vortexed for 10 s, and 2 μL were injected into the column.

Microdialysates were collected at a flow rate of 1 μL/min over 20 min, but the final volume possible to pipette varied between the samples (15 – 20 μL). Thus, the exact volume in each sample was measured with a pipette, transferred to the HPLC vial with a high recovery insert (Agilent, #5181–1270), and spiked with the IS solution in a 1:1 (v/v) ratio. The sample was mixed carefully and 5 μL were injected for analysis.

### 2.2. Instrumentation

#### 2.2.1. MS settings

Analysis was performed using an AB Sciex QTRAP 6500+ mass spectrometer (Framingham, MA, USA) coupled with an IonDrive Turbo V Ion Source. The source and gas parameters were as follows: nitrogen was used as a curtain and a collision gas, and it was set to 30 psi and medium level, respectively, while gases 1 and 2 (zero air) were both set to 50 psi. Genius 1024 generator (Peak Scientific; Billerica, MA, USA) provided for nitrogen and zero air. The source temperature was 450 °C, and the ion spray voltage was set to 4000 V. Analysis was performed under positive ion mode (ESI+) with multiple reaction monitoring (MRM) acquisition. The MRM transitions used for quantification were 294.1 → 170.0 (mass to charge ratio, *m/z*) for ondansetron and 297.1 → 173.1 (*m/z*) for ondansetron-D_3_ and the collision energy of 35 V was applied. The dwell time was 60 ms per transition.

#### 2.2.2. LC settings

The ExionLC AD system (AB Sciex, Framingham, MA, USA) was used for chromatographic separation, and analytes were eluted on a Gemini C18 column (5 μm; 50 × 4.6 mm) with a Gemini C18 guard cartridge (4 × 3.0 mm) (Phenomenex, Torrance, CA, USA). The column oven and the autosampler temperature were set to 40 °C and 15 °C, respectively. The mobile phases comprised 10 mM ammonium formate with 0.1 % formic acid in water for mobile phase A (MPA) and 0.1 % formic acid in acetonitrile for mobile phase B (MPB). The flow rate was set at 0.6 mL/min. The analytes were eluted under isocratic conditions with 45 % MPB and a total run time of 1.5 min. The mobile phase was diverted to waste for the first 0.5 min and the last 0.1 min of analysis. To reduce carryover, a mixture of isopropanol (IPA) and water (1:1, v/v) was injected every 4 samples under a W-shaped gradient condition set to the following: 45 % MPB (0 – 0.3 min), 45 → 95 % MPB (0.3 – 0.7 min), 95 % MPB (0.7 – 1.0 min), 95 → 10 % MPB (1.0 – 1.5 min), 10 → 95 % MPB (1.5 – 2.0 min), 95 % MPB (2.0 – 2.3 min), 95 → 10 % MPB (2.3 – 2.8 min), 10 → 95 % (2.8 – 3.3 min), 95 % MPB (3.3 – 3.6 min), 95 → 45 % (3.6 – 4.0 min), 45 % MPB (4.0 – 5.5 min). This gradient method for running IPA/water injections was diverted to waste. An external rinsing method was used to clean the needle before and after aspiration with a mixture of acetonitrile and water (1:1, v/v) with 0.1 % formic acid; then, additional external clean-up was applied by one-second rising with a mixture of methanol and water (20:80, v/v). The retention time for both ondansetron and ondansetron-D_3_ in serum was 0.89 min and in microdialysates was 0.93 min.

### 2.3. Method validation

This method was validated based on the guidelines from EMA [11] and FDA [12].

#### 2.3.1. Selectivity

Selectivity of the method was evaluated in six LOTs of blank rat serum, blank aCSF, and blank rat brain microdialysate to ensure that there were no interfering components from the matrix eluting at the retention times of ondansetron and the IS. The possible interfering peaks must be lower than 20 % of the analyte’s peak area, and 5 % of the internal standard’s peak area at the LLOQ level.

#### 2.3.2. Accuracy and precision

The within-run accuracy and precision were evaluated by 5 replicates for each QC level prepared on the same day. The between-run accuracy and precision were evaluated with 5 different runs for each QC level. Accuracy was calculated as a percent of the nominal concentration and precision as the percent relative standard deviation (%RSD). The accuracy was considered acceptable within 85–115 % of the nominal concentration, and %RSD was to not exceed 15 %. The LLOQ was defined as the lowest concentration measurable with accuracy within 80–120 % and %RSD ≤ 20 %.

#### 2.3.3. Stability tests

The stability of ondansetron in matrices and working solutions was assessed for low-QC and high-QC samples. QC samples were prepared in triplicates in rat blank serum and aCSF as a surrogate matrix for microdialysates. For assessing freeze–thaw stability, QC samples underwent 1 cycle of freezing (−80 °C) and thawing (room temperature, RT) for aCSF and 2 cycles for serum. For bench-top stability, QC samples were kept at RT for 2 h for aCSF and 4 h for serum. For long-term stability, QC samples were stored at −80 °C for 2 weeks for aCSF and 1 month for serum. To assess the stability of ondansetron in processed samples, QC samples were stored in the autosampler for 24 h at 15 °C. The stability of working solutions stored at −20 °C was tested at 4 months for microdialysis assays and at 5 months for serum. The ondansetron concentrations in QC samples were measured against freshly prepared calibrators. Analytes were considered stable if the concentrations were within 85–115 % of the nominal values.

#### 2.3.4. Matrix effect

A limited volume of animal brain microdialysates for experiments was noted, since collecting enough for preparing the calibrators and QC samples would require additional long microdialysis experiments in rats. Therefore, aCSF was evaluated as a surrogate matrix for preparing calibrators. The matrix effect was evaluated with 2 approaches based on the EMA [11] and newly released ICH guidelines [28]. Low- and high-QC samples were prepared in 6 different LOTs of rat serum, 6 individual rat brain microdialysates, 3 LOTs of aCSF, and water.

Per EMA guidelines [11], the matrix factors (MFs) were calculated by taking the ratio of the peak areas of compounds (ondansetron and IS) in the spiked matrix (serum, microdialysate, and aCSF) and their peak areas in spiked water. The IS-normalized MFs were further calculated by dividing the MFs of ondansetron and IS. The acceptable matrix effect was assumed if the IS-normalized MF was between 0.85 and 1.15, with %RSD ≤ 15 %.

ICH guidelines [28] require measuring the concentration of an analyte in each sample, assuming that a significant matrix effect would make the concentration fall outside the accuracy range. Thus, ondansetron concentration in each QC sample was calculated against a calibration curve prepared in aCSF (for microdialysate and aCSF) or serum (for serum samples). The calibration curves were prepared using a matrix from a single LOT. The accuracy of samples using various matrices should be within 85–115 % of the nominal concentrations and the precision (%RSD) < 15 % to rule out a significant matrix effect.

#### 2.3.5. Carryover

The presence of carryover was evaluated by analyzing blank samples injected after the highest calibrator. The peak areas at the retention time of the analyte and the IS were compared between the blank samples and the LLOQ samples injected during the same run. The signal in blank samples should not be greater than 20 % of the analyte response at the LLOQ level, and 5 % of the response for the IS.

### 2.4. Application of method

This method was used to quantify ondansetron in rat serum and brain microdialysis samples from a pilot study approved by the Rutgers University Institutional Animal Care and Use Committee (IACUC protocol 202000113). On day 0, two guide cannulas were implanted in the prefrontal cortex or the hippocampus of each rat. The procedure was performed on animals anesthetized with isoflurane at a concentration of 2 – 3 %, v/v. Microdialysis probes were inserted on day 7 and microdialysis study was performed on day 8 as follows: male Sprague Dawley rats (n = 4) were placed under anesthesia (isoflurane at a concentration of 1.5 – 3 %, v/v), and the microdialysis probes were perfused with aCSF at a flow rate of 1 μL/min. After 90 min of equilibration with aCSF, an IV bolus of 2 mg/kg ondansetron was administered via the jugular vein. Brain microdialysates were collected every 20 min for 3 h post-dosing, while blood samples (0.1 mL) were collected from the saphenous vein at different time intervals for 3 h post-dosing. Blood samples were centrifuged for 10 min at 15,700*g* at 4 °C, and serum was transferred to new tubes. All serum and microdialysis samples were stored at −80 °C until analysis.

## 3. Results

### 3.1. Method validation

#### 3.1.1. Selectivity

**Fig. 1.**
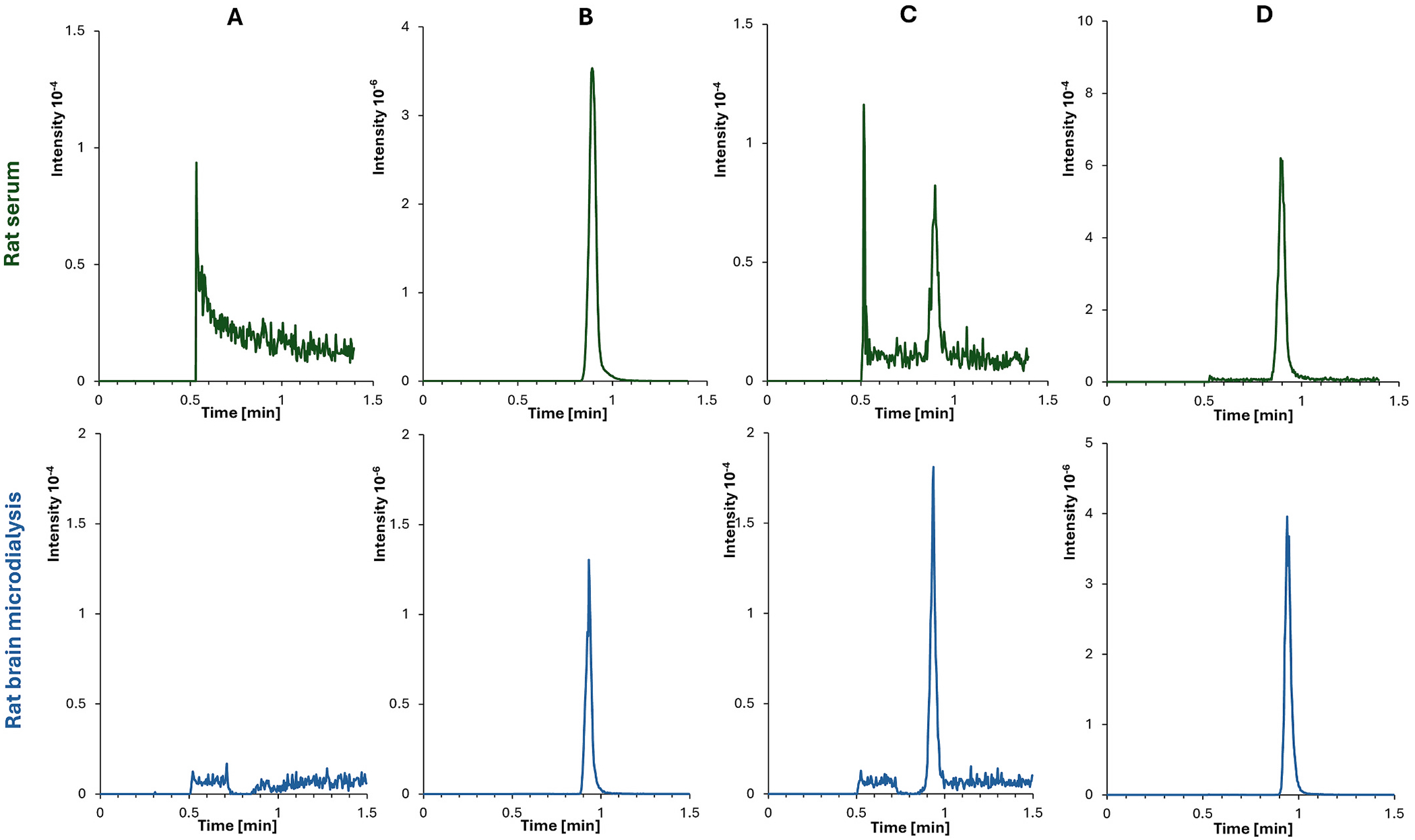
Representative chromatograms for rat serum and brain microdialysis assays: A) signal for ondansetron in blank samples, B) ondansetron-D_3_ in zero samples, C) ondansetron in LLOQ samples (0.01 μg/mL for serum and 0.025 ng/mL for aCSF), D) ondansetron in rat serum collected at 90 min with a concentration of 0.085 μg/mL after dosing ondansetron 2 mg/kg IV and rat brain microdialysate at 70 min with a concentration of 5.8 ng/mL.

No interfering peaks were present at the retention times of ondansetron and the IS in blank serum, aCSF, and blank microdialysate samples. The exemplary chromatograms of the blank and zero samples, LLOQ samples, and study samples from rats are presented in Fig. 1.

#### 3.1.2. Calibration curve

The method was linear in the concentration range of 0.010 to 25 μg/mL for serum and 0.025 to 50 ng/mL for microdialysates. The best fit was achieved for serum with linear regression and a weighting factor of 1/x^2^, and for microdialysates with power function and no weighting factor applied.

#### 3.1.3. Accuracy and precision

The results for within-run and between-run accuracy and precision are summarized in Table 1. For both matrices, the %RSD for LLOQ was below 20 % and accuracy was within 80–120 % of nominal concentrations, while for other concentration levels, it was below 15 % and within 85 – 115 %, respectively.

#### 3.1.4. Stability

The stability data for ondansetron in different matrices and under various storage conditions are shown in Table 2. Ondansetron was stable in rat serum after 2 cycles of freezing at −80 °C and thawing at RT and in aCSF after 1 cycle. The analyte was stable on the bench-top at RT for 4 h in serum and for 2 h in aCSF. Processed samples could be stored for at least 24 h in the autosampler at 15 °C without degradation. The analyte stability was verified in serum after 1 month and in aCSF after 2 weeks at −80 °C. Working solutions of ondansetron were stable when stored at −20 °C.

#### 3.1.5. Matrix effect

The results of the matrix effect experiment, performed according to the EMA guidelines [11], are presented in Table 3. There was a significant ion suppression for ondansetron in rat microdialysates (MFs of 69.8 – 71.9 %) and aCSF (MFs of 39.6 – 57.4 %). A similar pattern was observed for ondansetron-D_3_ (MFs of 68.6–70.8 % for microdialysates and 42.8 – 60.6 % for aCSF). There was no ion suppression either for ondansetron or IS in rat serum. The IS-normalized MFs for all matrices were close to 1.0, with %RSD < 15 % at both QC levels.

The results of the matrix effect experiment, performed according to the new ICH guidelines [28], are also presented in Table 3. The mean back-calculated concentrations were within 85–115 % of the nominal concentrations for rat microdialysates and serum.

#### 3.1.6. Carryover

Significant carryover was observed for ondansetron in serum samples, withthe response in blank sample 27 % of the LLOQ. Therefore, the additional washing samples were added in each analytical run; IPA/water samples (1:1, v/v) were injected with the W-shaped gradient conditions. It enabled to reduce the carryover in serum to 14 %. Moreover, the order of samples to be injected was controlled to minimize the carryover. In microdialysates, the mean carryover of 14 % was reduced to 9 % after implementing the above-mentioned washing samples. No carryover was observed for the IS either in serum or microdialysate.

### 3.2. In vivo pharmacokinetic study

This method was applied to a pilot intracerebral microdialysis study in rats to measure ondansetron concentrations in serum and brain microdialysates. For serum (μg/mL), the mean and the standard deviation (lowest to highest) of the minimum concentrations was 0.020 ± 0.012 (0.010 – 0.037) and of the maximum concentrations was 1.107 ± 0.072 (1.024 – 1.173) and for microdialysates (ng/mL) it was 0.88 ± 0.38 (0.43 – 1.39) for the minimum concentrations and 25.20 ± 12.80 (8.52 – 41.91) for the maximum concentrations. All concentrations were within the validated calibration range in both matrices.

## 4. Discussion

This bioanalytical assay was based on our previous LC-MS/MS method for ondansetron quantification in human CSF and plasma [17]. The method has been modified to account for the specific requirements of a microdialysis study (e.g., very low sample volume and analyte concentrations), which enabled the analysis of ondansetron concentrations in both rat serum and brain microdialysates. Our previous liquid–liquid extraction (LLE) procedure was replaced with simple protein precipitation to prepare serum samples. This allowed a higher throughput and faster processing times, enabling more study samples to be analyzed within a batch, compared to the LLE or solid phase extraction (SPE) techniques reported in literature [18–24]. In pharmacokinetic studies, which involve hundreds of samples to be analyzed, the method’s time and cost efficiency is a priority. The matrix volume for rat serum was also reduced by 10 times, from 25 μL of human plasma [17] to 2.5 μL of rat serum, which is crucial to permitting safe collection of multiple serum timepoints in rats. When collecting serial serum samples in rats the volume cannot exceed more than 10 % of the animals body weight, and hydration of the rat with subcutaneous saline is key to replenish their blood supply. Moreover, simpler sample preparation is in line with Green Analytical Chemistry approach and improves the method’s eco-friendliness as it allows for reducing the use of organic solvents and laboratory plastics. [29].

Although serum samples needed to be cleaned, collected microdialysates did not undergo a clean-up since these samples contained no or very low level of proteins [30,31]. Thus, brain microdialysates were injected into the column directly, after 1:1 dilution with the IS. The dilution step was necessary to obtain samples having the proportion of aqueous and organic solvents comparable to that of the mobile phase and thus to prevent the retention times from shifting. Importantly, a two-fold dilution did not affect the method’s accuracy. Injecting only 5 μL of sample enabled sufficient sensitivity to determine ondansetron concentrations in all microdialysates.

In our method, the calibration range for serum was wide and covered the concentrations from 0.010 to 25 μg/mL and for microdialysates it was 0.025 to 50 ng/mL. The previously published methods reported narrow ranges, which would not capture the full systemic concentration–time profiles in this microdialysis study [16–22]. Although Gaudette and colleagues [25] validated an LC-MS/MS method for quantifying ondansetron in 25 μL of rat plasma with an LLOQ of 0.02 ng/mL and in 100 μL of brain homogenates with an LLOQ of 0.002 ng/mL, this method was not validated for the microdialysate. Additionally, the sample volume was too large, which would not allow for frequent sampling in an animal. The calibration range reported by Duan and colleagues [24] was wider, however, their method was not fully validated and required 50 μL of plasma, which is an unacceptable volume when collecting at multiple timepoints in a single rat. The LLOQ of 0.025 ng/mL in microdialysates was the same as the LLOQ reported in our previously validated method in human CSF [17]. To our knowledge there are no other validated methods that have reported a higher sensitivity for ondansetron.

Initially, we could not reliably quantify ondansetron concentrations in microdialysates due to significant contamination of the blank microdialysates collected at baseline (before dosing). The source of contamination had to be identified and removed prior to continuing the microdialysis study. An in-depth inspection identified that ondansetron was adsorbing to glass syringes (or their metal pistons or rubber seals) that were used for both microdialysis and retrodialysis studies. An in vitro retrodialysis was performed to determine the recovery of ondansetron during brain microdialysis. The contamination in the glass syringes was resistant to sonication and cleaning with organic solvents (such as IPA, methanol, or acetonitrile). Since cross-contamination resulted from the re-use of microdialysis syringes, probes, and tubing adaptors, these had to be replaced for each study to prevent contamination in blank microdialysates. Adsorption of ondansetron to various materials such as metal and rubber can also explain the observed carryover. Carryover probably resulted from ondansetron adsorbing to the LC parts (e.g., needle, capillaries, seals). Previously, it was reported that ondansetron was adsorbing to the metal surfaces, and the extent of adsorption was directly proportional to the concentration of ondansetron [32]. A similar finding was observed for ondansetron adsorption to latex, which is the composition of rubber [33].

Given that it is not feasible to collect sufficient volumes of rat blank microdialysates for each assay, aCSF was used as a surrogate matrix to prepare calibrators and QC samples. Microdialysates were collected at a flow rate of 1 μL/min, so it would be very time-consuming to collect the volume required for several calibration curves. For example, collecting only 1 mL of blank microdialysate would take over 16 h. Moreover, the ethical reasons were just as important, with longer sample collection time puttting anesthesized rats at an increased risk of mortality. Using aCSF as the surrogate matrix eliminated the need to collect large volumes of rat blank microdialysates. The absolute and relative matrix effects [34] were absent in rat serum, which matches the findings of other ondansetron bioanalytical methods for plasma [17,18,21]. However, the absolute matrix effect was present in both rat brain microdialysates and aCSF, which was not the case in our previous method for human CSF, in which LLE was used to clean up the samples [17]. In both microdialysates and aCSF, there was a significant ion suppression, but the signals of ondansetron and IS were suppressed to the same extent, so the IS-normalized MFs were close to 1.0. Importantly, although there was almost twice larger ion suppression in aCSF compared to microdialysates (e.g., at low concentrations, MFs for ondansetron were 39.6 % and 71.9 %, respectively), due to the IS-normalized MFs not deviating from 1.0, the accuracies for the microdialysate QC samples read from the aCSF calibration curve were within the acceptable range (Table 3). Therefore, the experiment confirmed aCSF as a suitable surrogate matrix to replace brain microdialysate and the study samples could be analyzed against calibrators prepared in aCSF. Importantly, the matrix effect experiment revealed significant ion suppression in both microdialysate and aCSF. Although aCSF and microdialysate are considered relatively ‘clean’ matrices due to the lack or minimal content of proteins, they are rich in salts that contribute to ion suppression. The ion suppression could be reduced by modifying chromatographic conditions to prolong retention of ondansetron and separate the analyte from co-eluting salts or by adding the sample clean-up. However, it would negatively impact the method’s cost and time effectiveness, and its eco-friendliness due to higher use of solvents and laboratory materials. In our approach, the acceptable accuracies for brain microdialysate have been achieved by selecting an IS that corrected for the signal suppression of ondansetron. Thus, our example further emphasizes that a thorough method validation must be performed even for matrices that are considered relatively ‘clean’, and that limited validation tests might not be sufficient to confirm the method’s reliability.

## 5. Conclusions

An LC-MS/MS method was developed and validated to quantify ondansetron in rat serum and brain microdialysates. To our knowledge, this is the first fully validated method for the determination of ondansetron in rat brain microdialysates. The method is highly sensitive and requires only a low sample volume (15–20 μL for microdialysis and 2.5 μL for serum), so it fully meets the specific needs of the microdialysis study. Moreover, its simple sample preparation protocol enables a high throughput. The assay was successfully applied to an in vivo pilot microdialysis study to obtain the concentration–time profiles of ondansetron in rat serum and brain and will further be used in a larger cohort of rats to thoroughly assess the distribution of ondansetron into the brain.

## Figures and Tables

**Table 1:** Within-run and between-run precision and accuracy for ondansetron in rat serum and aCSF.

			Within-run (n = 5)		Between-run (n = 5)	
Matrix	QC level	Concentration	Precision (%RSD)	Accuracy (%)	Precision (%RSD)	Accuracy (%)
Serum	LLOQ	0.010 μg/mL	5.1	88.0	3.1	100.5
	Low	0.025 μg/mL	9.5	108.8	7.2	99.5
	Medium	0.5 μg/mL	3.6	98.6	1.6	98.0
	High	25 μg/mL	5.6	92.5	4.7	101.0
aCSF	LLOQ	0.025 ng/mL	12.9	117.6	7.4	103.1
	Low	0.050 ng/mL	2.3	98.8	6.1	103.9
	Medium	1 ng/mL	6.1	95.0	4.3	94.0
	High	50 ng/mL	7.7	112.3	4.0	104.6

LLOQ, lower limit of quantitation; QC, quality control; %RSD, percent relative standard deviation.

**Table 2:** Stability of ondansetron in rat serum and aCSF.

		Matrix								Working solutions	
SERUM	concentration (n = 3)	freeze–thaw		bench-top		autosampler		long-term		long-term (5 months, −20 °C)	
		(2 cycles, −80 °C)		(4 h, RT)		(24 h, 15 °C)		(1 month, −80 °C)			
						
		mean accuracy (%)	RSD (%)	mean accuracy (%)	RSD (%)	mean accuracy (%)	RSD (%)	mean accuracy (%)	RSD (%)	mean accuracy (%)	RSD (%)

Low-QC	0.025 μg/mL	113.3	4.1	105.3	2.2	113.6	4.0	105.3	5.8	94.7	2.4
High-QC	25 μg/mL	94.1	2.6	93.1	2.3	95.9	4.4	89.9	4.4	94.1	4.8
aCSF	concentration (n = 3)	freeze–thaw (1 cycle, −80 °C)		bench-top (2 h, RT)		autosampler (24 h, 15 °C)		long-term (2 weeks, −80 °C)		long-term (5 months, −20 °C)	
		mean accuracy (%)	RSD (%)	mean accuracy (%)	RSD (%)	mean accuracy (%)	RSD (%)	mean accuracy (%)	RSD (%)	mean accuracy (%)	RSD (%)
Low-QC	0.050 ng/mL	92.0	2.2	106.7	2.9	97.3	3.1	91.3	2.5	92.0	2.2
High-QC	50 ng/mL	89.2	3.2	99.2	5.6	97.9	4.0	87.8	2.6	102.6	9.4

aCSF, artificial cerebrospinal fluid; QC, quality control; RSD, relative standard deviation; RT, room temperature.

**Table 3:** Matrix effect results for ondansetron in rat brain microdialysates, aCSF, and rat serum based on EMA and ICH guidelines.

			EMA guidelines				ICH guidelines	
Matrix	Concentration	N	Ondansetron MF	Ondansetron-D_3_ MF	IS-normalized MF		Accuracy (%)	%RSD
			(mean ± SD)%	(mean ± SD)%	(mean ± SD)	%RSD		
Rat microdialysates	0.05 ng/mL	6	71.9 ± 5.9	68.6 ± 6.4	1.05 ± 0.03	3.3	109.0	3.4
	50 ng/mL	6	69.8 ± 6.3	70.8 ± 8.0	0.99 ± 0.05	5.4	107.9	5.4
aCSF	0.05 ng/mL	3	39.6 ± 9.7	42.8 ± 8.7	0.92 ± 0.05	5.5	95.3	4.8
	50 ng/mL	3	57.4 ± 18.2	60.6 ± 23.4	0.97 ± 0.11	11.6	106.3	11.7
Rat serum	0.025 μg/mL	6	103.2 ± 3.5	103.1 ± 3.1	1.00 ± 0.02	2.0	106.0	2.1
	25 μg/mL	6	96.3 ± 3.3	100.7 ± 0.9	0.96 ± 0.03	2.8	89.5	2.8

aCSF, artificial CSF; IS, internal standard; MF, matrix factor; SD, standard deviation; %RSD, percent relative standard deviation.

## Data Availability

Data will be made available on request.

## Abbreviations:

aCSF: artificial cerebrospinal fluid
CSF: cerebrospinal fluid
EMA: European Medicines Agency
FDA: Food and Drug Administration
high-QC: quality control at high concentration level
HPLC-UV: high-performance liquid chromatography method with ultraviolet detection
IPA: isopropanol
IS: internal standard
LC-MS/MS: liquid chromatography-tandem mass spectrometry
LLE: liquid–liquid extraction
LLOQ: lower limit of quantification
low-QC: quality control at low concentration level
MF: matrix factor
mid-QC: quality control at medium concentration level
MPA: mobile phase A
MPB: mobile phase B
MRM: multiple reaction monitoring
Pgp: P-glycoprotein
QC: quality control
RSD: relative standard deviation
RT: room temperature
SPE: solid phase extraction

## References

[1] Lockwood S, Dickenson AH, What goes up must come down: insights from studies on descending controls acting on spinal pain processing, J. Neural Transm 127 (2020) 541–549, 10.1007/s00702-019-02077-x. 31515656 PMC7148257

[2] Scott JA, Wood M, Flood P, The Pronociceptive Effect of Ondansetron in the Setting of P-Glycoprotein Inhibition, Anesth. Analg 103 (2006) 742, 10.1213/01.ane.0000228861.80314.22. 16931690

[3] Chiang M, Back H, Lee JB, Oh S, Guo T, Girgis S, Park C, Haroutounian S, Kagan L, Pharmacokinetic Modeling of the Impact of P-glycoprotein on Ondansetron Disposition in the Central Nervous System, Pharm. Res 37 (2020) 205, 10.1007/s11095-020-02929-2. 32989520 PMC8752326

[4] Hammarlund-Udenaes M, Microdialysis in CNS PKPD Research: Unraveling Unbound Concentrations, in: Müller M (Ed.), Microdialysis Drug Dev, Springer, New York, NY, 2013: pp. 83–102. 10.1007/978-1-4614-4815-0_5.

[5] de Lange EC, de Boer AG, Breimer DD, Methodological issues in microdialysis sampling for pharmacokinetic studies, Adv. Drug Deliv. Rev 45 (2000) 125–148, 10.1016/s0169-409x(00)00107-1. 11108971

[6] Chefer VI, Thompson AC, Zapata A, Shippenberg TS, Overview of Brain Microdialysis, Curr. Protoc. Neurosci. Editor. Board Jacqueline N Crawley Al CHAPTER (2009) Unit7.1. 10.1002/0471142301.ns0701s47. PMC2953244 19340812

[7] Chen C, Zhou H, Guan C, Zhang H, Li Y, Jiang X, Dong Z, Tao Y, Du J, Wang, Zhang T, Du N, Guo J, Wu Y, Song Z, Luan H, Wang Y, Du H, Zhang C. Li, Chang H, Wang T, Applicability of free drug hypothesis to drugs with good membrane permeability that are not efflux transporter substrates: A microdialysis study in rats, Pharmacol. Res. Perspect 8 (2020) e00575, 10.1002/prp2.575. 32266794 PMC7138916

[8] Wang S, Chen C, Guan C, Qiu L, Zhang L, Zhang S, Zhou H, Du H, Li C, Wu Y, Chang H, Wang T, Effects of membrane transport activity and cell metabolism on the unbound drug concentrations in the skeletal muscle and liver of drugs: A microdialysis study in rats, Pharmacol. Res. Perspect 9 (2021) e00879, 10.1002/prp2.879. 34628723 PMC8502442

[9] Tsai T-H, Liu M-C, Determination of unbound theophylline in rat blood and brain by microdialysis and liquid chromatography, J. Chromatogr. A 1032 (2004) 97–101, 10.1016/j.chroma.2003.09.009. 15065783

[10] Tsai T-H, Concurrent measurement of unbound genistein in the blood, brain and bile of anesthetized rats using microdialysis and its pharmacokinetic application, J. Chromatogr. A 1073 (2005) 317–322, 10.1016/j.chroma.2004.10.048. 15909536

[11] European Medicines Agency, Guideline on Bioanalytical Method Validation, (2011). http://www.ema.europa.eu/docs/en_GB/document_library/Scientific_guideline/2011/08/WC500109686.pdf. 10.4155/bio.12.44 22533559

[12] Food and Drug Administration, Bioanalytical Method Validation Guidance for Industry, (2018). https://www.fda.gov/files/drugs/published/Bioanalytical-Method-Validation-Guidance-for-Industry.pdf.

[13] Cen Y, Shan Y, Zhao J, Xu X, Nie Z, Zhang J, Multiple drug transporters contribute to the brain transfer of levofloxacin, CNS Neurosci. Ther 29 (2023) 445–457, 10.1111/cns.13989. 36253925 PMC9804084

[14] Hurtado FK, Weber B, Derendorf H, Hochhaus G, Dalla Costa T, Population Pharmacokinetic Modeling of the Unbound Levofloxacin Concentrations in Rat Plasma and Prostate Tissue Measured by Microdialysis, Antimicrob. Agents Chemother 58 (2014) 678–686, 10.1128/aac.01884-13. 24217697 PMC3910848

[15] Siemiątkowska A, Kagan L, New biological matrix – Full method validation: Exaggeration or necessity? A case study with tariquidar, J. Chromatogr. B 1228 (2023) 123842, 10.1016/j.jchromb.2023.123842. 37524013

[16] Chong YE, Chiang M, Deshpande K, Haroutounian S, Kagan L, Lee JB, Simultaneous quantification of ondansetron and tariquidar in rat and human plasma using a high performance liquid chromatography-ultraviolet method, Biomed. Chromatogr 33 (2019) e4653. 31322284 10.1002/bmc.4653 PMC6800589

[17] Siemiątkowska A, Frey K, Gurba KN, Crock LW, Haroutounian S, Kagan L, An LC-ESI-MS/MS method for determination of ondansetron in low-volume plasma and cerebrospinal fluid: Method development, validation, and clinical application, J. Pharm. Biomed. Anal 235 (2023) 115625, 10.1016/j.pba.2023.115625. 37549552 PMC10529361

[18] Ninama G, Patel R, Patel M, Shah G, Solid phase extraction liquid chromatography mass spectrometry method with electrospray ionization for the determination of Ondansetron in human plasma: Development and validation consideration, Arab. J. Chem 10 (2017) S3135–S3141, 10.1016/j.arabjc.2013.12.004.

[19] Moreira RF, Salvadori MC, Azevedo CP, Oliveira-Silva D, Borges DC, Moreno RA, Sverdloff CE, Borges NC, Development and validation of a rapid and sensitive LC-ESI-MS/MS method for ondansetron quantification in human plasma and its application in comparative bioavailability study, Biomed. Chromatogr 24 (2010) 1220–1227, 10.1002/bmc.1431. 20954214

[20] Dotsikas Y, Kousoulos C, Tsatsou G, Loukas YL, Development and validation of a rapid 96-well format based liquid–liquid extraction and liquid chromatography–tandem mass spectrometry analysis method for ondansetron in human plasma, J. Chromatogr. B 836 (2006) 79–82, 10.1016/j.jchromb.2006.03.032. 16581316

[21] Alvarez J-C, Charbit B, Grassin-Delyle S, Demolis J-L, Funck-Brentano C, Abe E, Human plasma quantification of droperidol and ondansetron used in preventing postoperative nausea and vomiting with a LC/ESI/MS/MS method, J. Chromatogr. B 879 (2011) 186–190, 10.1016/j.jchromb.2010.12.001. 21185793

[22] Liu K, Dai X, Zhong D, Chen X, Quantitative determination of ondansetron in human plasma by enantioselective liquid chromatography-tandem mass spectrometry, J. Chromatogr. B 864 (2008) 129–136, 10.1016/j.jchromb.2008.02.002. 18299256

[23] Bauer S, Störmer E, Kaiser R, Tremblay P-B, Brockmöller J, Roots I, Simultaneous determination of ondansetron and tropisetron in human plasma using HPLC with UV detection, Biomed. Chromatogr 16 (2002) 187–190, 10.1002/bmc.125. 11920943

[24] Duan M, Zhao Q, Zhong D, Yuan Y, Pharmacokinetics of R-(−)ondansetron compared with that of S-(−)ondansetron in rats using an LC–MS/MS method, Biomed. Chromatogr 33 (2019) e4426. 30408206 10.1002/bmc.4426

[25] Gaudette F, Bédard D, Kwan C, Frouni I, Hamadjida A, Beaudry F, Huot P, Highly sensitive HPLC-MS/MS assay for the quantitation of ondansetron in rat plasma and rat brain tissue homogenate following administration of a very low subcutaneous dose, J. Pharm. Biomed. Anal 175 (2019) 112766, 10.1016/j.jpba.2019.07.014. 31330277

[26] Hakkarainen JJ, Jalkanen AJ, Kääriäinen TM, Keski-Rahkonen P, Venäläinen T, Hokkanen J, Mönkkönen J, Suhonen M, Forsberg MM, Comparison of *in vitro* cell models in predicting *in vivo* brain entry of drugs, Int. J. Pharm 402 (2010) 27–36, 10.1016/j.ijpharm.2010.09.016. 20920560

[27] Ding P, Xu H, Wei G, Zheng J, Microdialysis sampling coupled to HPLC for transdermal delivery study of ondansetron hydrochloride in rats, Biomed. Chromatogr 14 (2000) 141–143, 10.1002/1099-0801(200005)14:3<141::AID-BMC937>3.0.CO;2-Z. 10850615

[28] International Council for Harmonisation of Technical, Requirements for Pharmaceuticals for Human Use, ICH guideline M10 on bioanalytical method validation and study sample analysis, (2022). https://www.ema.europa.eu/en/documents/scientific-guideline/ich-guideline-m10-bioanalytical-method-validation-step-5_en.pdf.

[29] Locatelli M, Kabir A, Perrucci M, Ulusoy S, Ulusoy HI, Ali I, Green profile tools: Current status and future perspectives, Adv. Sample Prep 6 (2023) 100068, 10.1016/j.sampre.2023.100068.

[30] Carreño F, Helfer VE, Staudt KJ, Olivo LB, Barreto F, Herrmann AP, Rates SMK, Costa T. Dalla, Quantification of neurotransmitters in microdialysate samples following quetiapine dosing to schizophrenia phenotyped rats using a validated LC-MS/MS method, J. Chromatogr. B 1155 (2020) 122282, 10.1016/j.jchromb.2020.122282. 32771966

[31] Davies MI, Cooper JD, Desmond SS, Lunte CE, Lunte SM, Analytical considerations for microdialysis sampling, Adv. Drug Deliv. Rev 45 (2000) 169–188, 10.1016/S0169-409X(00)00114-9. 11108973

[32] Vengatesh G, Karthik G, Sundaravadivelu M, A comprehensive study of ondansetron hydrochloride drug as a green corrosion inhibitor for mild steel in 1 M HCl medium, Egypt. J. Pet 26 (2017) 705–719, 10.1016/j.ejpe.2016.10.011.

[33] Ruiz A, Llácer JM, Morales E, Gallardo V, Physical characteristics of polymer complexes in suspension obtained from cellulosic latexes with ondansetron, J. Mater. Sci. Mater. Med 15 (2004) 659–664, 10.1023/B:JMSM.0000030206.55732.f3. 15346732

[34] Matuszewski BK, Constanzer ML, Chavez-Eng CM, Strategies for the Assessment of Matrix Effect in Quantitative Bioanalytical Methods Based on HPLC–MS/MS, Anal. Chem 75 (2003) 3019–3030, 10.1021/ac020361s. 12964746

